# Soluble Wood Smoke Extract Promotes Barrier Dysfunction in Alveolar Epithelial Cells through a MAPK Signaling Pathway

**DOI:** 10.1038/s41598-019-46400-8

**Published:** 2019-07-11

**Authors:** Matthew R. Zeglinski, Christopher T. Turner, Rui Zeng, Carley Schwartz, Stephanie Santacruz, Megan A. Pawluk, Hongyan Zhao, Arthur W. H. Chan, Christopher Carlsten, David J. Granville

**Affiliations:** 1 International Collaboration on Repair Discoveries (ICORD), Vancouver Coastal Health Research Institute, University of British Columbia (UBC), Vancouver, BC Canada; 2 Department of Pathology and Laboratory Medicine, UBC, Vancouver, BC Canada; 3 UBC Centre for Heart Lung Innovation, St. Paul’s Hospital, UBC, Vancouver, BC Canada; 4 British Columbia Professional Firefighters’ Burn and Wound Healing Group, Vancouver, BC Canada; 5 Department of Medicine, Division of Respiratory Medicine, Chan-Yeung Centre for Occupational and Environmental Respiratory Disease, Vancouver Coastal Health Research Institute, UBC, Vancouver, BC Canada; 6 Department of Chemical Engineering & Applied Chemistry, University of Toronto, Toronto, ON Canada

**Keywords:** Cell signalling, Mechanisms of disease, Respiration, Respiratory tract diseases

## Abstract

Wildfire smoke induces acute pulmonary distress and is of particular concern to risk groups such as the sick and elderly. Wood smoke (WS) contains many of the same toxic compounds as those found in cigarette smoke (CS) including polycyclic aromatic hydrocarbons, carbon monoxide, and free radicals. CS is a well-established risk factor for respiratory diseases such as asthma and COPD. Limited studies investigating the biological effects of WS on the airway epithelium have been performed. Using a cell culture-based model, we assessed the effects of a WS-infused solution on alveolar epithelial barrier function, cell migration, and survival. The average geometric mean of particles in the WS was 178 nm. GC/MS analysis of the WS solution identified phenolic and cellulosic compounds. WS exposure resulted in a significant reduction in barrier function, which peaked after 24 hours of continuous exposure. The junctional protein E-cadherin showed a prominent reduction in response to increasing concentrations of WS. Furthermore, WS significantly repressed cell migration following injury to the cell monolayer. There was no difference in cell viability following WS exposure. Mechanistically, WS exposure induced activation of the p44/42, but not p38, MAPK signaling pathway, and inhibition of p44/42 phosphorylation prevented the disruption of barrier function and loss of E-cadherin staining. Thus, WS may contribute to the breakdown of alveolar structure and function through a p44/42 MAPK-dependent pathway and may lead to the development and/or exacerbation of respiratory pathologies with chronic exposure.

## Introduction

Chronic obstructive pulmonary disease (COPD) is a progressive disorder that affects approximately 251 million people worldwide and accounted for 5% of all-cause mortality in 2015^1^. Although cigarette smoke (CS) is the leading cause of COPD, other exposures including occupational dusts and fumes, indoor and outdoor air pollution (*eg*, diesel exhaust and wood smoke (WS)) have been linked to increased incidence and exacerbation of COPD^1^. In particular, the impact of WS on respiratory health has become a growing area of research due to both the increase in the size and number of annual forest fires in many parts of the world and a growing appreciation of the effects of WS on exposed individuals such as first responders. Smoke generated during forest fires not only affects local communities but also, due to weather patterns, can travel great distances and settle in communities in different regions.

Akin to CS, WS contains thousands of compounds and generates high concentrations of particulate matter (PM). While the exact chemical composition of WS is not known, selected analysis has shown WS to contain high levels of carbon monoxide, nitrogen oxides, dioxins, and polycyclic aromatic hydrocarbons associated with PM, all of which have been shown to have a negative effect on respiratory health^2,3^. Collectively, these chemicals and PM induce local inflammation^4^ and mediate cytotoxicity and oxidative stress^2,5^.

In response to smoke exposure, dysregulated intracellular signaling pathways have a profound effect on cell function and phenotype. Specifically, the aryl hydrocarbon receptor (AhR) is activated in response to dioxin and dioxin-like compounds found in both CS and WS that, when bound to a ligand, translocates to the nucleus and binds to dioxin response elements to alter gene expression^6^. AhR has been shown to enhance cytochrome P450 1A1 (CYP1A1), which, in addition to its detoxifying role, can generate mutagenic metabolites and ROS leading to further cell stress^6–8^. CS also activates the epidermal growth factor receptor (EGFR) leading to focal adhesion kinase (FAK) and Src activation, TGF-β_1_ production, and induction of epithelial-mesenchymal transition (EMT)^9^. Additionally, EGFR stimulation by CS has been shown to induce hypoxia inducible factor 1α (HIF-1α) through PI3K and Erk1/2 (p44/42) mitogen-activated protein kinase (MAPK) signaling pathways^10^. Inhibition of this pathway at the level of the receptor and/or kinase prevents HIF-1α expression and mucus formation in CS exposed airways^10^.

In the current study, WS was found to disrupt epithelial barrier structure and function through the loss of cell-cell adhesion and impairs the wound healing capacity of alveolar epithelial cells after injury. WS exposure also promoted induction of the p44/42, but not p38, MAPK signaling pathway. Moreover, p44/42 phosphorylation correlated with the breakdown of cell-cell adhesions and loss of epithelial barrier function which could, in part, be prevented by blocking p44/42 phosphorylation.

## Results

### The effect of wood smoke on cell viability

**Figure 1:**
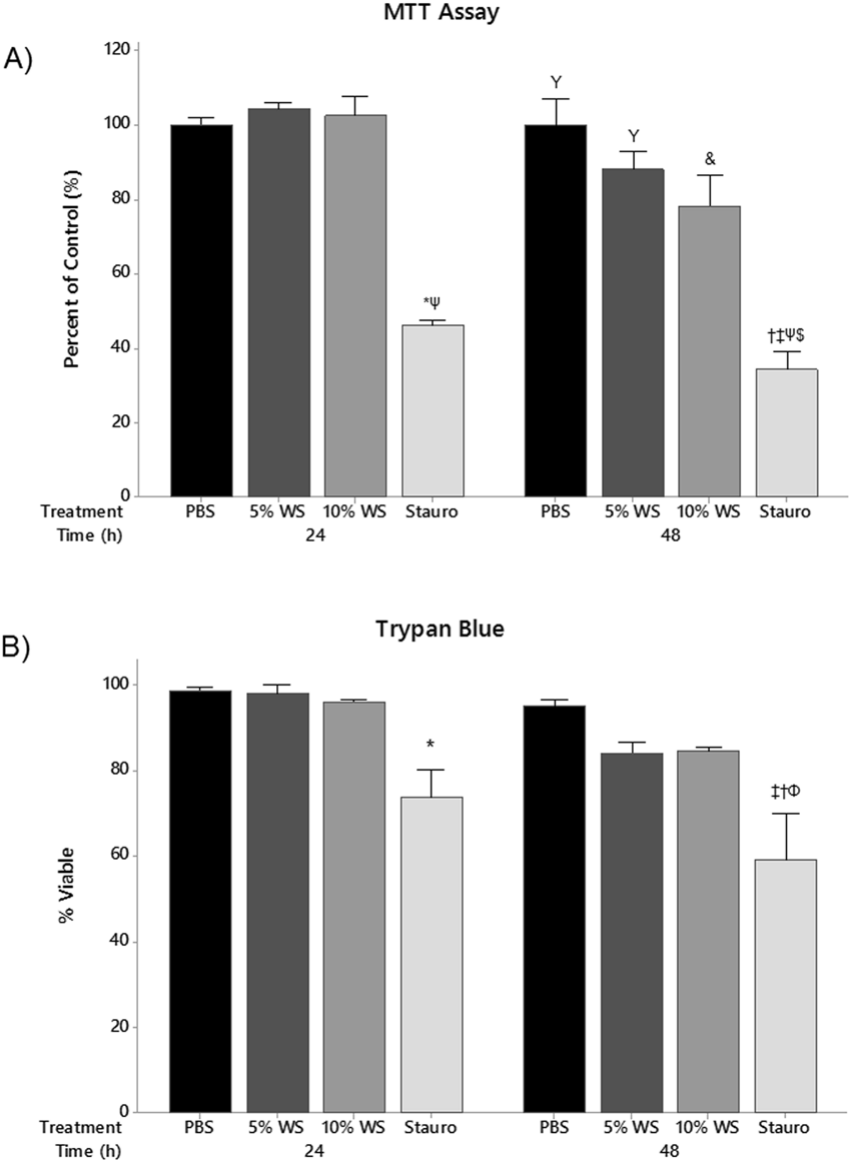
Wood Smoke and Cell Viability. (**A**) MTT and (**B**) trypan blue exclusion were used to assess the effects of WS on A549 cell viability. Staurosporine was used as a positive control for reduced viability. MTT *P < *0*.*001* vs. 24 h 5% WS, 10% WS; ^†^*P* < *0*.*001* vs. 24 h 10% WS, ^‡^*P* < *0*.*001* vs. 24 h 5% WS, ^Ψ^*P* < *0*.*001* vs. 24 h PBS, ^&^*P* < *0*.*01* vs. 24 h Stauro, ^Υ^*P* < *0*.*001* vs. 24 h Stauro, ^$^*P* < *0*.*001* vs. 48 h PBS, 5% WS, 10% WS; n = 3). Trypan blue exclusion (*P < *0*.*05* vs. 24 h Unt and 24 h 5% WS; ^†^*P* < *0*.*001* vs. 24 h Unt; ^‡^*P* < *0*.*001* vs. 24 h 5% and 10% WS and 48 h Unt; ^Φ^*P* < *0*.*05* vs. 48 h 5% and 10% WS; n = 3). Statistical differences were determined using a two-way ANOVA followed by a Tukey’s post-hoc test. Data presented as mean ± se.

To assess whether WS has an effect on epithelial viability, MTT and trypan blue methods were employed. There was no reduction in cell viability with up to 10% WS exposure after 24 hours (Fig. 1A,B). There was a trend of reduced viability after 48 hours of exposure with increasing concentrations of WS, however it was not significant. Cells treated with staurosporine, a broad spectrum protein kinase inhibitor that promotes cell death, were used as a positive control for reduced cell viability. Staurosporine resulted in a significant reduction in cell viability as compared to WS exposure after 24 and 48 hours. There was no difference in viability between 24 and 48 hour staurosporine treatments.

### Characterization of wood smoke particulate

**Figure 2:**
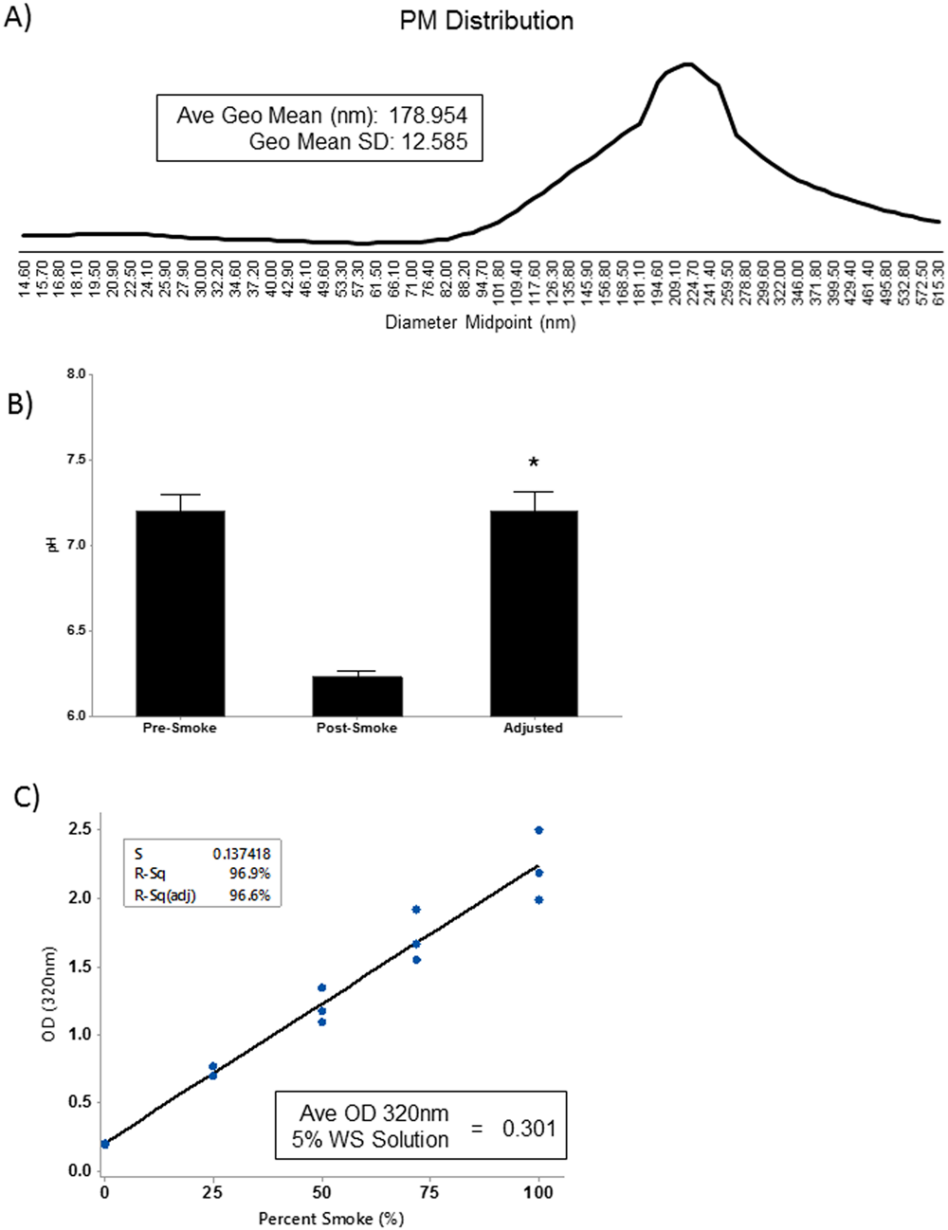
Characterization of Birch Wood Smoke and the Wood Smoke Infused Solution. (**A**) A representative distribution of the PM generated (nm) from the combustion of birch wood. The average geometric mean was 178.954 ± 12.585 nm. Data presented as mean ± SD. (**B**) The pH for PBS was assessed prior to, and following, WS infusion. pH of the resulting solution was adjusted to that prior to WS infusion. *P < *0*.*001* vs. pre-smoke and adjusted; n = 3. Statistical difference was determined by one way ANOVA followed by a Tukey’s post-hoc test. (**C**) Generation of a standard curve was accomplished by assessing the absorbance of the solution at 320 nm. Repeated measurements of WS infused PBS generated a highly reproducible curve (R^2^ = 96.8%). The average OD320 of a 5% wood smoke solution was 0.301. R-squared was determined by linear regression.

The distribution of PM generated during combustion was determined using a TSI Model 3936 Scanning Mobility Particle Sizer. A distribution profile of all PM represented in the WS was generated and the average geometric mean of the PM determined. Combustion of 1.0 gram of birch wood generated PM with an average geometric mean of 178.954 ± 12.585 nm (Fig. 2A). There was an overall greater abundance of PM >100 nm compared to PM <100 nm.

### Characterization of wood smoke generated solutions

The pH of the WS-generated solutions dropped significantly (*P* < 0.05) from 7.1 at baseline to 6.0 following smoke infusion (Fig. 2B). pH was adjusted to baseline using NaOH. To limit variability between experiments, a standard curve was generated from the WS solution by analyzing the OD 320 nm as described previously for CS studies^11^. Linear regression demonstrates a highly reproducible smoke solution (R-squared = 96.9%) between three individual experiments (Fig. 2C). As we found that a concentration range of 0–10% WS did not induce cytotoxicity, we focused on the effects of 5–10% WS solutions for the rest of the paper.

**Figure 3:**
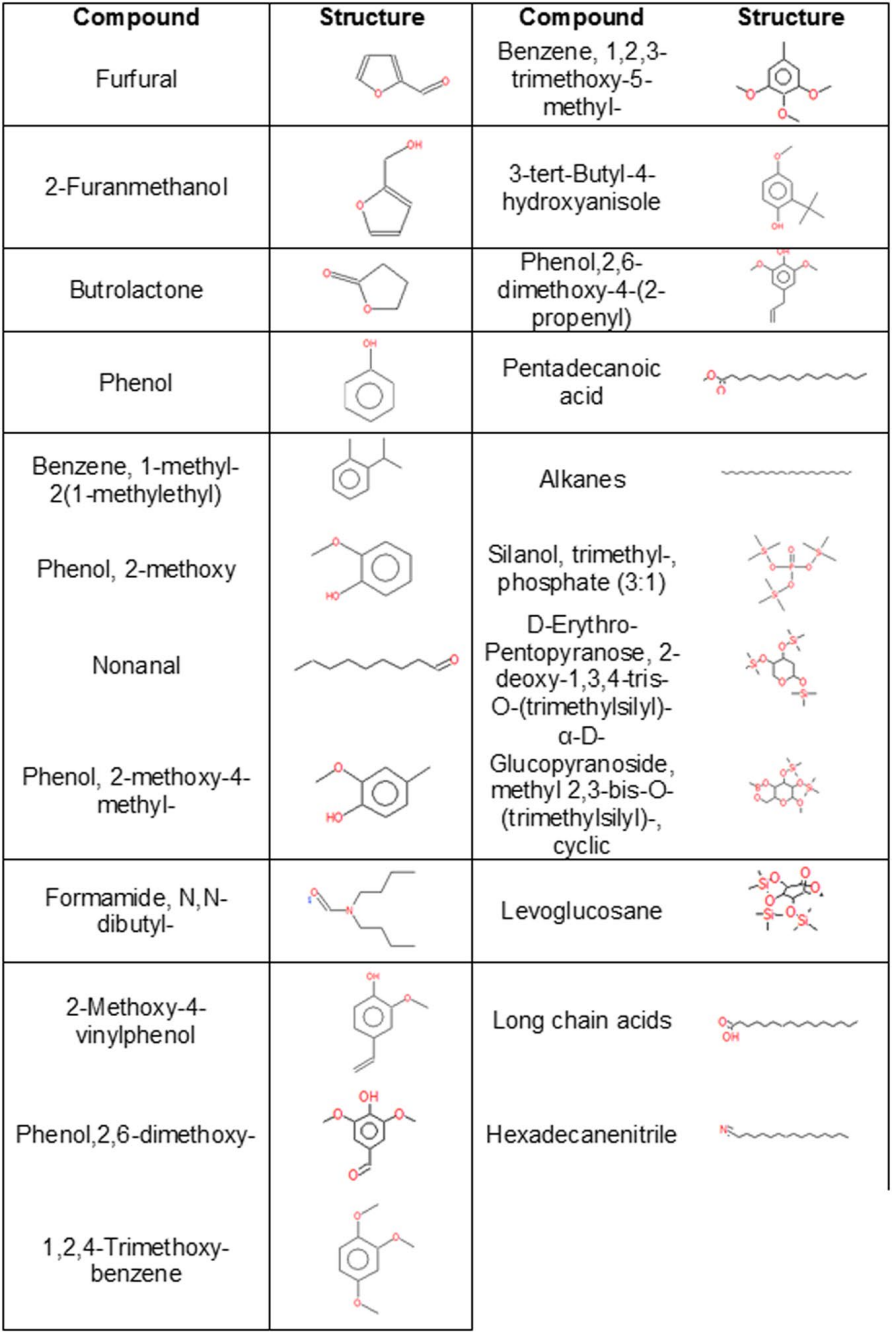
GC/MS Analysis of Dissolved Wood Smoke Compounds. Structural analysis of identified compounds dissolved in the generation of wood smoke solution.

GC/MS was performed on two separate WS solutions to identify aqueous-dissolved compounds. We identified a number of methoxyphenols, benzenes, alkanes, and cellulosic compounds (i.e. levoglucosan) within our WS solution. A complete list of the compounds identified and their structures are provided in Fig. 3.

### Wood smoke reduces epithelial barrier function

**Figure 4:**
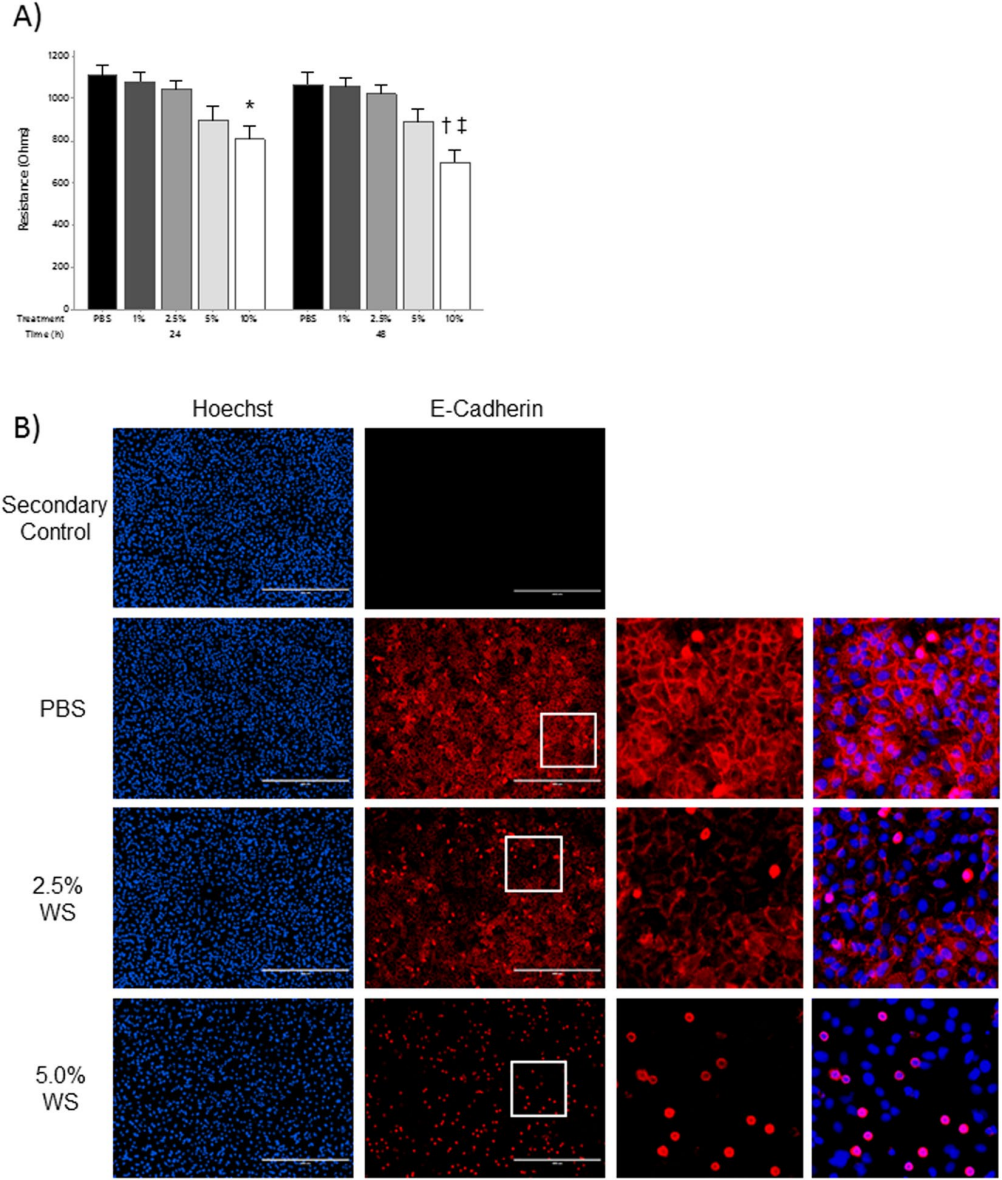
Wood Smoke and Epithelial Barrier Function. (**A**) Epithelial barrier function was evaluated at 24 and 48 hours by ECIS. Resistance at 4 kHz was used as a measure of barrier function. *P < *0*.*05* vs. 24 h PBS, 1% WS; ^†^*P* < *0*.*001* vs. 24 h PBS; ^‡^*P* < *0*.*01* vs. 24 h 1% WS, 2.5% WS and 48 h PBS, 1% WS 2.5% WS; n = 3. Statistical differences were determined using a two-way ANOVA and a Tukey’s post-hoc test. Data presented as mean ± se. (**B**) Cell-cell contacts were assessed 24 hours post-WS exposure *via* staining for the cell adhesion marker E-cadherin. Immunofluorescent staining demonstrated a dose dependent loss of typical cobblestone E-cadherin staining and accumulation of intense intracellular staining. Cell coverage was demonstrated by staining nuclei with Hoechst (scale bar = 400 μm; n = 3).

CS impairs the epithelial barrier of the airways and lungs^12^. Therefore, we set out to determine whether WS has a similar effect on the function and integrity of A549 alveolar epithelial cell barrier function. Using electrical cell-substrate impedance sensing (ECIS) we continuously monitored the epithelial barrier following exposure to a range of WS concentrations. Concentrations up to 5% WS showed no change in epithelial resistance at 4 kHz (measure of barrier) at either the 24 or 48 hour time points as compared to PBS control (Fig. 4A). However, 10% WS exposure for 24 and 48 hours demonstrated a significant reduction in epithelial barrier function (Fig. 4A). There was no difference in epithelial resistance associated with 10% WS exposures at 24 versus 48 hours.

To assess the structural integrity of the epithelial barrier, cells were grown to confluence, exposed to WS, and stained for the intercellular adhesion junction protein, E-cadherin. PBS treated cells demonstrated typical cobblestone patterning of E-cadherin in confluent monolayers suggesting an intact barrier (Fig. 4B). Exposure to 2.5% WS for 24 hours resulted in a reduction of E-cadherin staining intensity and a loss of the cobble stone patterning which was completely absent with 5% WS exposure.

### Wood smoke inhibits epithelial migration

**Figure 5:**
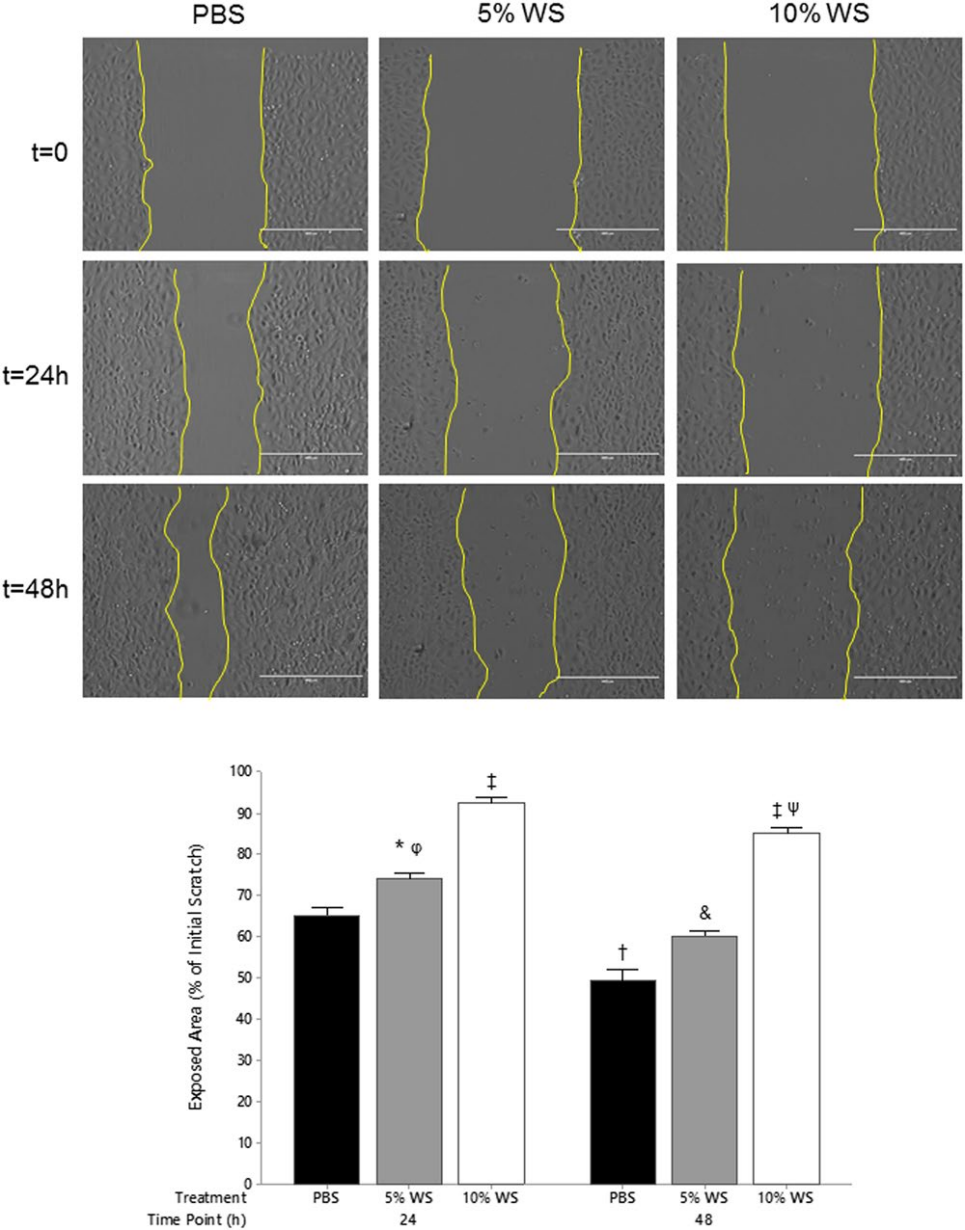
Impaired Migration following Wood Smoke Exposure. A549 cells were grown to confluent monolayers, scratched, and exposed to 5% and 10% WS for up to 48 hours. *P < *0*.*05* vs. 24 h 5% WS; ^†^*P* < *0*.*001* vs. 24 h PBS; ^‡^*P* < *0*.*001* vs. 24 h and 48 h PBS, 24 h 5% WS, 48 h 5% WS; ^&^*P* < *0*.*01* vs. 24 h 5% WS, 48 h PBS; ^φ^*P* < *0*.*001* vs. 48 h PBS; ^Ψ^*P* < *0*.*01* vs. 24 h 5% WS; n = 3. Statistical differences were determined using a two-way ANOVA followed by a Tukey’s post-hoc test. Data presented as mean ± se.

To assess the wound healing capacity of epithelial cells following WS exposure, confluent monolayers were wounded (scratched) and monitored for 48 hours. Within 24 hours of the initial injury, PBS treated cells had reduced the open area by >30% (Fig. 5). By 48 hours, PBS treated cells had further reduced the open area by an additional 20% (50% total). There was a significant difference between 5% (*P* < 0.05) and 10% (*P* < 0.001) WS compared to PBS after 24 hours which was also seen after 48 hours. Those cells exposed to 24 hours of 10% WS had significantly impaired migration, having closed <10% of the original injured area (Fig. 5). This trend continued out to the 48 hour time point where cells exposed to 10% WS had closed <20% of the original wounded area compared to 5% WS exposure where >40% of the original wound area had been closed.

### Activation of MAPK signaling pathways following wood smoke exposure

**Figure 6:**
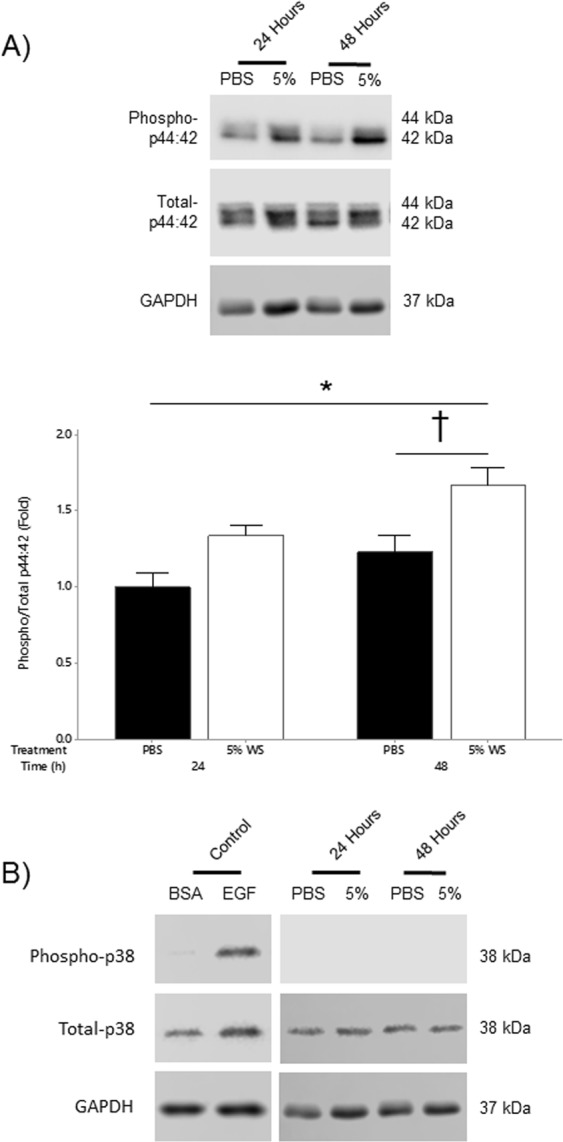
Activation of MAPK Signaling following Wood Smoke Exposure. (**A**) WS exposure resulted in activation of a p44/42 dependent MAPK signaling pathway. After 24 hours of WS exposure there was approximately a 50% increase in p44/42 phosphorylation although this was not significant. After 48 hours, there was a significant increase in p44/42 phosphorylation as compared to untreated cells after both 24 and 48 hours (*P < *0*.*05* vs. 24 h Unt; ^†^*P* < *0*.*05* vs. 48 h Unt; n = 4). (**B**) There was no evidence of p38 phosphorylation after 24 or 48 hours of exposure. EGF (10 ng/mL) stimulated cells were used as a positive control. BSA treated cells were used as a control for EGF stimulation. Images were cropped to show a representative image of each treatment and time point. All images were derived from the same blot following stripping and re-probing. Full blots are provided in Supplementary Figs S1, S2 and S3. Statistical differences were determined using a two-way ANOVA followed by a Tukey’s post-hoc test. Data presented as mean ± se.

CS and diesel exhaust promote activation of MAPK signaling pathways^9,13^. We postulated that WS exposure in alveolar epithelial cells would induce similar intracellular signaling pathways. Western blot analysis shows a non-significant trend to increased phosphorylation of p44/42 after 24 hours of WS exposure. However, there was a significant (*P* < 0.05) increase in phospho-p44/42 levels after 48 hours of 5% WS exposure (Fig. 6A). There was no evidence of p38 phosphorylation at either the 24 or 48 hour time points (Fig. 6B).

### Inhibition of p44/42 signaling does not effect cell viability and improves the epithelial barrier

**Figure 7:**
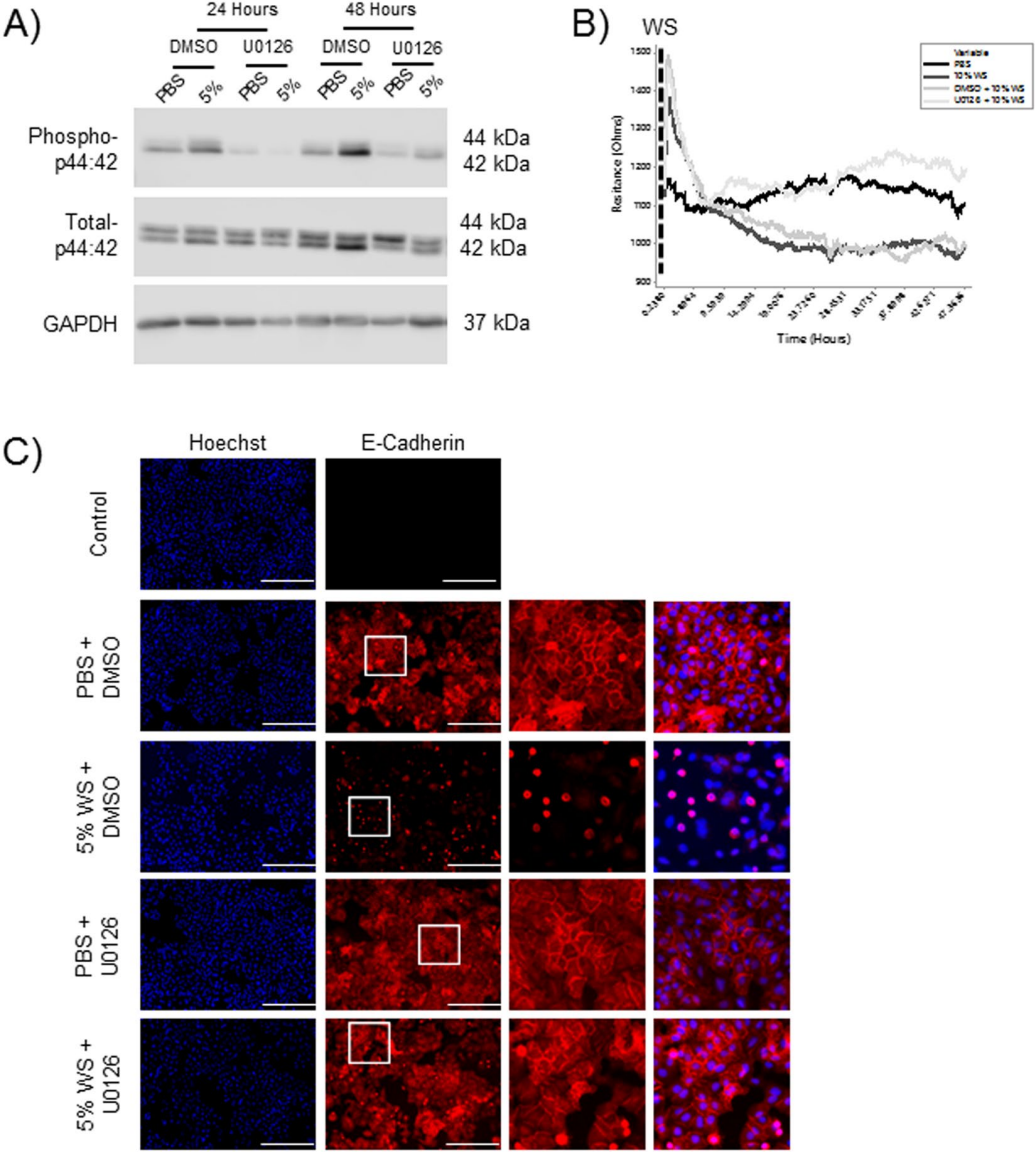
U0126 Prevents Wood Smoke Mediated Epithelial Dysfunction. Pre-treatment of A549 cells with 10 µM U0126 for 60 minutes prior to WS exposure prevented (**A**) p44/42 phosphorylation and (**B**) a reduction in epithelial resistance. (Dashed line – start of wood smoke treatment). (**C**) 24 hours of WS exposure resulted in the loss of membrane E-cadherin staining and cellular accumulation. However, pre-treatment with U0126 partially prevented loss of membrane E-cadherin staining as characterized by the preserved cobble stone patterning (scale bars = 400 µm; n = 3). Full blots are provided in Supplementary Fig. S4.

**Figure 8:**
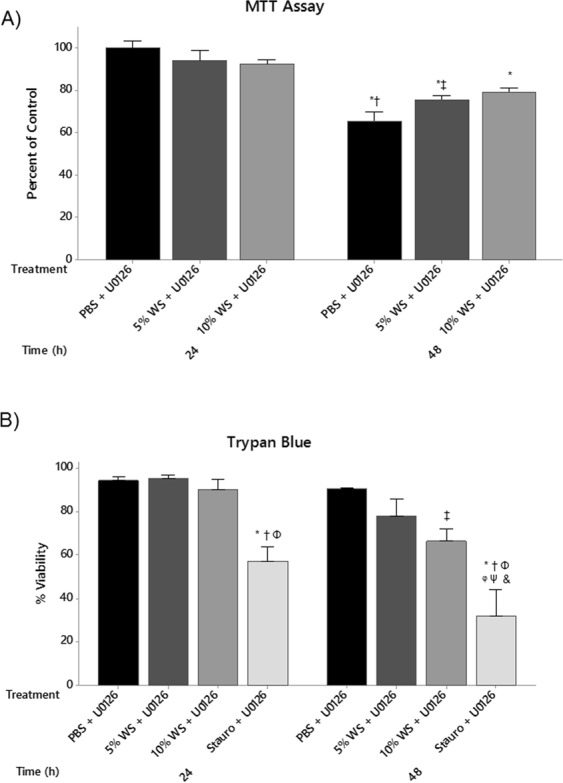
U0126 Pre-Treatment Does Not Reduce Cell Viability. (**A**) MTT and (**B**) trypan blue exclusion were used to assess the effects of U0126 and wood smoke on A549 cell viability. Neither assay detected a reduction in cell viability among wood smoke exposed groups. Staurosporine was used as a positive control for reduced viability. MTT (*P < *0*.*01* vs. 24 h Untreated; ^†^*P* < *0*.*001* vs. 24 h 5% WS + U0126 and 24 h 10% WS + U0126; ^‡^*P* < *0*.*05* vs. 24 h 5% WS + U0126 and 24 h 10% WS + U0126). Trypan blue exclusion (*P < *0*.*01* vs. 24 h Unt + U0126; ^†^*P* < *0*.*01* vs. 24 h 5% WS + U0126; ^‡^*P* < *0*.*05* vs. 24 h 5% WS + U0126; ^Φ^*P* < *0*.*01* vs. 24 h 10%WS + U0126; ^φ^*P* < *0*.*05* vs. 48 h 10% WS + U0126; ^Ψ^*P* < *0*.*01* vs. 48 h 5% WS + U0126; ^&^*P* < *0*.*01* vs. 48 h Unt + U0126; n = 3). Statistical differences were determined using a two-way ANOVA followed by a Tukey’s post-hoc test. Data presented as mean ± se.

As activation of the p44/42 MAPK signaling pathway following WS exposure appears to correlate with reduced epithelial barrier integrity/function, we wanted to know whether blocking p44/42 MAPK signaling could prevent and/or reduce the observed loss in epithelial integrity following WS exposure. Confluent monolayers were grown and pre-treated with 10 μM U0126 60 minutes prior to WS exposure. Western blot analysis demonstrated that U0126 efficiently prevented p44/42 phosphorylation at both the 24 and 48 hour time points (Fig. 7A). ECIS analysis of the epithelial barrier demonstrated preserved barrier function with U0126 pre-treatment prior to exposure to WS (Fig. 7B), lending additional support for a role in p44/42 MAPK signaling in WS-mediated barrier dysfunction. In support of the results reported in Fig. 4B, there was a significant loss of E-cadherin patterning at 24 hours in 5% WS cells as compared to PBS control (Fig. 7C). U0126 pre-treatment did not affect the cobblestone E-cadherin patterning in PBS treated cells. Those cells pretreated with U0126 prior to 5% WS exposure had evidence of preserved cobblestone E-cadherin staining throughout the monolayer (Fig. 7C). Notably, U0126 pre-treatment did not affect cell viability when used in combination with WS exposures at either 24 or 48 hours as determined by MTT assay and trypan blue exclusion (Fig. 8). Staurosporine treated cells showed a significant reduction in cell viability (*P* < 0.05).

## Discussion

Cigarette smoking is associated with continuous cycles of injury, inflammation, and repair which results in increased permeability, senescence, and cell death of the airway epithelium^14–16^. While the effects of CS on airway and alveolar epithelial cells are well established^17,18^, studies into the effects of WS on the alveolar epithelium are lacking. In the current study, we have provided evidence that WS results in a dose-dependent reduction of epithelial barrier function, and cell migration without affecting cell viability, which appears to be mediated through the p44/42 MAPK signaling pathway.

WS cytotoxicity has been reported with conflicting results. Exposure of A549 cells to PM_0.1–10_ generated from the combustion of a mix of hardwoods and softwoods demonstrated a significant increase in LDH release after 40 hours of exposure as compared to controls^3^. Similarly, A549 cells exposed to PM_1_ from either birch, beech (hardwoods), or spruce (softwood) showed a significant reduction in cell viability as determined by MTT assay and PI exclusion^2^. Conversely, rat alveolar epithelial type II cells exposed to WS extract for 24 hours displayed a significant increase in cell viability as determined by an MTT assay^19^. These later results are similar to those observed in our current study. We observed no significant difference in cell viability as determined by either MTT or trypan blue exclusion after 24 or 48 hours of WS exposure. Our results may differ from those of the former studies due to the type of WS/source exposure. Additionally, in the current study, we generated a WS-infused solution that includes both particles and water-soluble gases. This is in contrast to those solutions used in previous studies that contained only PM generated from the combustion of different wood sources. Moreover, these studies used PM of a single size or a limited range that would further limit the findings. PM is a major inducer of respiratory inflammation and barrier disruption^20,21^. It is possible that the concentration of PM in our WS-infused solution is different than that of previous studies. However, these previous studies do not examine the effect that noxious and toxic gases generated during combustion would have on barrier function and integrity, whereas we have included both dissolved gases and PM in our assessment of barrier function; although the precise composition of our WS solution is unknown, it likely more closely mimics the exposure environment experienced by the alveoli *in vivo* and may be a more translationally relevant model to assess the effects of WS on alveolar epithelium.

The alveolar epithelium forms a physical and functional barrier to the external environment as it plays a critical role in gas exchange, preventing exogenous insults from entering the blood stream, and promoting an immune response. Cadherins are a major structural component of epithelial adhesion junctions that anchor cells to one another and plays an important role in cell signaling^22^. Loss of E-cadherin is associated with a loss of epithelial barrier function and increased permeability. Exposure to CS extract is associated with the loss of airway structure and function. Human bronchial epithelial cells exposed to 1–5% CS extract show a reduction in E-cadherin staining and a significant loss of barrier function^12,23–25^. A549 cells exposed to 2.5% CS extract show a loss of E-cadherin mRNA and protein expression, as well as the cell adhesion marker ZO-1^24,26^. In addition to CS, WS contributes to the disruption of the respiratory epithelium^27,28^. Smoke generated from the combustion of China fir significantly reduced E-cadherin staining in rat tracheal epithelial cells and modestly reduced staining in small airways of rat tissues following smoke exposure^27^. A study comparing firefighter instructors and non-smokers demonstrated that chronic exposure to WS alters the permeability of the alveolar-capillary barrier to ^99m^TcDTPA (Technetium TC-99M diethylene triamine pentaacetic acid, a radionucleotide chelate complex)^28^. The negative effect of WS on the alveolar epithelial barrier in our study is in agreement with those previously reported. Real-time monitoring of the epithelial barrier using ECIS found that WS significantly reduced epithelial barrier function in a dose dependent manner. Reduction of the epithelial barrier correlated with a loss of membrane E-cadherin staining, suggesting that WS plays a role in the breakdown of cell-cell adhesion and may play a role in increased permeability.

CS and its extracts promote cell migration of JEG-3 human placental cells^29^ and gingival fibroblasts^30^. Human bronchial epithelial cells exposed to four hours of CS demonstrated a marked increase in cell migration compared to controls^31^. Similar to CS, studies suggest that WS can alter cell migration. Lung fibroblasts exposed to WS generated from the combustion of English oak show a significant increase in fibronectin expression, suggesting that cell migration may also be enhanced^32^. In contrast to these previous reports on migration in CS and WS, we found that WS exposure of A549 cells inhibited cell migration in a dose dependent manner (Fig. 5). Exposure to 10% WS for 48 hours demonstrated less than 20% wound closure compared to 50% closure seen in controls. This opposite effect compared to that seen with CS studies may be a result of the additional compounds that are found in CS as a result of the chemical processing of cigarettes during manufacturing. Although one study demonstrated that WS promoted fibronectin expression^32^, and thus may promote cell migration, fibroblast migration was not directly assessed limiting the scope of the study’s findings.

CS and WS exposure results in activation of numerous intracellular signaling pathways that affect cell function. In particular, MAPK signaling has been reported to be activated following both CS and WS exposure. CS exposure of human bronchial epithelial cells results in robust p38 phosphorylation within 60 minutes of stimulation^33,34^ and inhibition of p44/42 phosphorylation has been shown to blunt the effects of smoke exposure^34^. Furthermore, both CS and WS exposure in human lung fibroblasts have shown dose-dependent increases in p44/42 phosphorylation^32^. PM_2.5_ generated from combustion of China fir promotes p44/42 phosphorylation in primary human airway epithelial cells and NCI-H292 cells (human lung epithelial cells) in a EGFR dependent manner^35^. BEAS-2B cells (transformed human bronchial epithelial cells) exposed to WS particulate leads to increased phosphorylation of both p44/42 and p38^36^. These previous studies both support and contrast the results of the current study. We found that WS exposure specifically increased p44/42 phosphorylation and had no effect on p38 activation (Fig. 6). Using U0126, a potent and specific inhibitor of p44/42, we were able to prevent the loss in membrane E-cadherin expression, suggesting that p44/42 inhibition was able to maintain epithelial barrier integrity following wood smoke exposure (Fig. 7). It was surprising to see no activation of p38 signaling following WS exposure. Lack of p38 phosphorylation could be due to differences in the type of exposure between studies. Previous studies that reported p38 phosphorylation focused on the use of PM as opposed to whole smoke extract^33,34,36^. Increasing the PM exposure in our model may generate results that would mimic those of previous studies. It is also possible that p38 phosphorylation happens rapidly following WS exposure and the signal is transient such that it is not present after 24 hours. Previous reports have shown that p38 phosphorylation occurs within minutes of EGF stimulus and diminishes overtime^37–39^. This could explain the lack of p38 activation in our study. A temporal assessment of the MAPK signaling pathways following WS exposure is warranted to characterize these events.

There are several limitations to our study that merit discussion. First, we generated WS by combusting birch wood. Birch was chosen as it is abundant in the northern regions of North America, including those areas that are prone to forest fires. Birch is a hardwood which tends to burn longer and hotter than softwoods. Softwoods emit a greater concentration of PM_2.5_ and generate more CO_2_ than hardwoods^40^. Conversely, hardwoods produce more CO and NOx compared to softwoods^40^. Particulate composition can vary depending on several factors including burn temperature, air supply, amount of wood, wood moisture, and burn time^41^. Therefore, the choice of combustible material and intensity of combustion can have a significant effect on the distribution and types of compounds that are emitted during the burning process. We were able to generate a PM profile of our WS and found that the average geometric size of the PM was ~170 nm. A distribution profile (Fig. 2A) demonstrated that there was a greater amount of PM >100 nm shifting the curve to the right. This range is similar to that previously reported for combustion of oak (another hardwood) where the majority of PM generated was greater than 100 nm^42^. A second study demonstrated that hot burning combustion of hardwoods generated PM that was primarily <600 nm^42^. Although these results are similar to those that we observed, it is difficult to draw a direct comparison as in this study PM was reported based on a range of sizes (i.e. 300–600 nm) rather than individual sizes as we have done^41^. Taken together our results are comparable to those previously reported for other hardwood combustions, however the role of softwoods in our system is unknown.

A second limitation is that we are only examining the effects of aqueous-soluble WS compounds and a portion of PM on alveolar epithelial cells. Although bubbling smoke into solutions for treatment of cell cultures is common practice^11,43,44^, this process may not represent all of the compounds found in WS as gases that do not dissolve in aqueous solutions are lost during the infusing process. In an attempt to describe those compounds that were dissolved in our WS solution, we performed GC/MS on WS infused solutions. We identified numerous compounds that have previously been identified in other WS studies including methoxyphenols and cellulosic compounds such as levoglucosan (Fig. 3)^45,46^. Levoglucosan is generally considered non-toxic however its presence in biofluids, such as urine, has been suggested as a biomarker for WS exposure and thus exposure to more toxic compounds such as polyaromatic hydrocarbons^47^. However, levoglucosan has a short half-life in the body whereby >70% of it has been excreted within 7 hours of exposure^47^. Guaiacol, a methoxyphenol compound, is produced through the pyrolysis of lignin and has also been proposed as a marker of WS exposure. However, unlike levoglucosan, guaiacol may be carcinogenic as it can enhance cell proliferatation and has been found in the urine of patients with neuroblastoma^48^. Interestingly, guaiacol has been identified as a potent antioxidant^49^ with good reactive species scavenging potential. The relevance of this scavenger activity in the current study is unknown. Additional compounds identified that are likely to play a role in epithelial health include benzenes, alkanes, and long chain acids (Fig. 3).

A third limitation is that *in vivo* alveolar cells are not submerged, *per se*, and will be exposed to gases and particulate in a manner that differs from the current methodology. Culturing cells at an air-liquid interface may be a more relevant model; however, without specialized equipment, exposing the cells to smoke and ensuring the reproducibility of each exposure is technically difficult^50^. Finally, we did not differentiate between the effects of PM and dissolved gases in our exposure system. It is possible that the loss of barrier function may be the result of particulate exposure whereas p44/42 intracellular signaling cascades may be in response to dissolved gases. Further study is required to tease out the contribution of each factor to the responses that we observed in our system.

## Methods

### Cell culture

A549 cells were grown and maintained in complete Dulbecco’s Modified Eagle’s media (DMEM, Gibco) supplemented with 10% fetal bovine serum (FBS, Gibco) and 100 U/mL penicillin/streptomycin (Gibco). Cells between passages 3–8 were used for all experiments.

### Analysis of wood smoke particulate matter

Approximately 1.0 gram of natural birch wood toothpicks (Polar Pak) were ignited and 60-cc of the smoke collected using a syringe. This procedure was repeated 3x (for a total of 240-cc of smoke analyzed). A TSI Model 3936 Scanning Mobility Particle Sizer (SMPS) was used to measure the electrical size mobility distribution of particles in the size range from 14 nm to 670 nm. The particle sizer consists of two components: (1) the Model 3775 condensation particle counter, and (2) the Series 3080 electrostatic classifier. Scanning times of 120 seconds with a retrace of 15 seconds were performed every 3 minutes. The geometric mean mobility diameter (and its geometric standard deviation (SD)) were recorded.

### Generation of wood smoke-infused solution

Generation of WS-infused PBS was conducted as previously described with modifications^11^. Briefly, approximately 1.0 gram of natural birch wood toothpicks (Polar Pak) was ignited, the smoke collected and gently bubbled through 5.0 mL of phosphate buffered saline (PBS) using a 60 mL syringe and 2.4 mm (internal diameter) silicon tubbing (Tygon), over the course of 1 minute. This procedure was repeated 3 times for a total of 4 minutes. pH of the resulting solution was determined using the Accumet AB15 (Fisher) pH meter and was adjusted to 7.1 using 0.2 M NaOH. The final solution was not filtered, so as to mimic the particulate insult experienced by the lungs *in vivo*. This solution was considered to be a 100% WS infused solution. From this, a dilution series (0–100%) in PBS was performed and a standard curve generated by reading its absorbance at 320 nm (TECAN Infinite M1000 Pro, TECAN). This curve served as a reference point for each smoking experiment to help correct for variance in the OD of WS infused PBS in each smoking event. The smoke solution was used immediately and the remaining solution discarded. A fresh WS solution was made for each new experimental exposure. PBS controls had room air infused at the same rate and duration as that of WS infused solutions.

### Gas chromatography/mass spectrophotometry of wood smoke infused solution

The composition of the WS solution was analyzed by thermal desorption gas chromatography mass spectrometry^51^. 3 µL of WS solution was injected onto a small prebaked quartz filter punch (4 mm diameter). The punch was then placed in the thermal desorption system (TDS3, Gerstel) and thermally desorbed in helium at 300 °C for 5 min. The desorbed analytes were first pre-concentrated on a quartz liner and then desorbed at 300 °C into a GC/MS (7890B/5977A, Agilent). The GC column used was Rxi-5ms (Restek) with dimensions of 30 m length × 0.25 mm ID and a film thickness of 0.25 µm. The column was held at 50 °C for 5 min, and ramped at 10 °C/min to 300 °C and held for 2 min. Eluted compounds are ionized by electron impact at 70 eV, and identified by matching to NIST08 mass spectral library.

### Electrical cell-substrate impedance sensing (ECIS)

Each well of an 8W10E array (Applied Biophysics) was coated in 0.2 M cysteine for 15 minutes at room temperature. The wells were washed twice in ultrapure water before addition of complete cell culture medium. Arrays were stabilized using the ECIS Zθ system (Applied Biophysics) as recommended by the manufacturer before the addition of cells. A549 cells were seeded at a density of 2 × 10^5^ cells per well. The array was placed in an incubator and the ECIS system started on multi-frequency (62.4–64000 Hz) reading. Once the cells had formed a confluent monolayer and generated a stable barrier (as determined by ECIS measurements at 64 kHz and 4 kHz, respectively), cells were washed twice in PBS and serum-starved for 4 hours before being exposed to the WS-infused solution. ECIS resistance readings were continually monitored in real time for 48 hours.

### Cell viability

A549 cells were seeded into each well of a 12-well dish and grown to confluence (~2 days). Cells were washed with PBS, serum-starved for 3 hours, and then treated with DMSO (vehicle; Sigma) or 10 μM U0126 (Cell Signaling) for an additional hour prior to WS exposure. *MTT Assay*: 24 and 48 hours after WS exposure, 100 μL of MTT solution (5.0 mg/mL, Sigma) was added to each well and incubated for 3 hours at 37 °C. Culture media was then removed and formazan crystals dissolved in 1.0 mL of a 50% DMSO, 50% ethanol solution. 200 μL of the resulting solution was added to each well of a 96-well dish and absorbance read at 570 nm (TECAN Infinite M1000 Pro, TECAN). *Trypan Blue Exclusion*: 24 and 48 hours after WS exposure, culture media was removed and placed in a 1.5 mL microtube. Cell monolayers were washed in PBS and the wash was added to the culture media. Adherent cells were removed from the plate using 0.25% trypsin-EDTA (Gibco) and added to the collected media and wash. Cells were pelleted at 600 *xg* for 5 minutes at room temperature. Supernatant was removed and discarded. Cell pellets were re-suspended in 1.0 mL of PBS. An equal volume of cell suspension and 0.4% trypan blue solution (Sigma) were incubated together at room temperature for 5 minutes. 10 μL of the resulting solution was added to each chamber of an automated cell counting chip and the number of viable cells was determined using the BioRad TC20 cell counter.

### Western blot

A549 cells (2.5 × 10^5^) were seeded into 6-cm cell culture dishes and grown to confluence. Cells were washed and serum-starved prior to WS exposure as described above. Cells were lysed in CelLytic MT (Sigma) containing protease (Sigma) and phosphatase (Sigma) inhibitors. Lysates were incubated on ice for 15 minutes followed by sonication. Cell debris was pelleted by centrifugation at 18,000 × *g* for 20 minutes at 4 °C. Supernatant (cell lysate) was decanted and protein concentration determined by BCA (Thermo). 15 μg of cell lysate was separated by 10% SDS-PAGE at 150 volts for 80 minutes and proteins transferred to 0.45 μm PVDF membrane at 300 mA for 75 minutes on ice. Blots were washed once in 0.2% TBS-T and blocked for 60 minutes at room temperature in 5% skim milk TBS-T. Blots were washed twice in TBS-T and incubated overnight at 4 °C in primary antibodies: phospho-p44/42 (Cell Signaling; 5% BSA TBS-T, 1:1000, RRID: AB_331646), p44/42 (Cell Signaling; 5% skim milk TBS-T, 1:1000, RRID: AB_330744), phospho-p38 (Cell Signaling; 5% BSA TBS-T, 1:1000, RRID: AB_331296), p38 (Cell Signaling; 5% skim milk TBS-T, 1:1000, RRID: AB_330713), GAPDH (Cell Signaling; 5% BSA TBS-T, 1:1000, RRID: AB_561053). The next day, blots were washed 3x and incubated for 60 minutes at room temperature with secondary antibody: goat-anti-rabbit-HRP (RRID: AB_2313567) or goat-anti-mouse-HRP (RRID: AB_10015289) (Jackson Labs, 3% skim milk TBS-T, 1:3333). Blots were imaged using ECL substrate (Thermo) on the Licor Odyssey Fc system under the chemiluminescence channel. Images were saved and analyzed using Quantity One (BioRad). No adjustments were made to the image and no post-analysis modifications to the data were made (i.e. background correction). Bands were manually traced and the density/mm^2^ was recorded. Blots were probed for phosphorylated proteins first, were stripped in 0.2 M NaOH for 45 minutes, and re-probed as described above for total proteins. The stripping and re-probing process was repeated once more and blots re-probed for GAPDH loading control. Epidermal growth factor (EGF, 10 ng/mL, Lonza) treated cells were used as a positive control for activation of MAPK signaling pathways. Images are representative of all proteins from the same blots following stripping and re-probing.

### Immunofluorescence

A549 cells (2.5 × 10^5^) were seeded into 6-cm cell culture dishes with coverslips and grown to confluence. Cells were washed and serum starved prior to WS exposure as described above. Cells were fixed in 4% paraformaldehyde for 15 minutes and permeabilized in 0.1% Triton X-100 PBS for an additional 15 minutes. Cells were gently washed 3x in PBS and blocked in 10% donkey serum PBS for 60 minutes at 37 °C. Cells were then washed and incubated overnight at 4 °C with primary antibody E-Cadherin (R&D; 1% donkey serum PBS, 2.0 μg/mL, RRID: AB_355568). The next day, primary antibody was removed, cells washed, and donkey anti-goat 594 secondary antibody (Life Technologies; 1% donkey serum PBS; 1:700) was added for 60 minutes at room temperature in the dark. Cells were washed and nuclei stained with Hoechst solution (1:10000 in PBS) for 2 minutes at room temperature. Cells were then washed twice in PBS and once in distilled water. Coverslips were mounted to slides using Hydromount (Electron Microscopy Sciences). Slides were imaged using the EVOS FL Cell Imaging System (Life Technologies) under 10x magnification. Images were merged and analyzed using Photoshop CS6 software (Adobe). Images for control and treatment groups were collected at the same time under the same conditions.

### Cell migration – scratch assay

A549 cells were seeded into 6-cm cell culture dishes and grown to confluence. Cells were washed twice in PBS and 2% DMEM-FBS was added to each well. The cell monolayers were scratched using a 20–200 micropipette tip creating a (+) sign to help with orientation for repeated measurements. Scratches were imaged on the EVOS FL at 20x magnification. This was considered t = 0. The culture media was removed to remove cell debris from scratching the monolayer, fresh 2% DMEM-FBS added to each well, and cells exposed to WS. Plates were incubated at 37 °C for up to 48 hours following the initial scratch. Monolayers were imaged every 24 hours, with migration determined by measuring the scratched area at t = 0, 24, and 48 hours using ImageJ software.

### Statistics

All data is presented as mean ± standard error. A two-way ANOVA with a Tukey’s post-hoc test was performed for all dose + temporal WS studies. A *P*-value less than 0.05 (*P < *0*.*05*) was considered statistically significant. Data was analyzed using Minitab 17 software.

## Supplementary information

Dataset 1
