# Supplementary material for: Soluble Wood Smoke Extract Promotes Barrier Dysfunction in Alveolar Epithelial Cells through a MAPK Signaling Pathway
Source: Sci Rep. 2019 Jul 11;9:10027. doi: 10.1038/s41598-019-46400-8 (PMC6624307; doi:10.1038/s41598-019-46400-8)
Supplement: Supplementary file 1 — Dataset 1 [file 41598_2019_46400_MOESM1_ESM.pdf]

Soluble Wood Smoke Extract Promotes Barrier Dysfunction in Alveolar Epithelial Cells through a MAPK Signaling Pathway

Matthew R. Zeglinski[1,2,3,4], Christopher T. Turner[1,2,3,4], Rui Zeng[6], Carley Schwartz[3,5], Stephanie Santacruz[1,2,3,4], Megan A. Pawluk[1], Hongyan Zhao[1,2,3,4], Arthur W. H. Chan[6], Christopher Carlsten[3,5], David J. Granville[1,2,3,4*]

[1]International Collaboration on Repair Discoveries (ICORD), Vancouver Coastal Health Research Institute, University of British Columbia (UBC), Vancouver, BC, Canada
[2]Department of Pathology and Laboratory Medicine, University of British Columbia, Vancouver, BC, Canada
[3]UBC Centre for Heart Lung Innovation, St. Paul's Hospital, UBC, Vancouver, BC, Canada
[4]British Columbia Professional Firefighters' Burn and Wound Healing Group, Vancouver, BC, Canada
[5]Department of Medicine, Division of Respiratory Medicine, Chan-Yeung Centre for Occupational and Environmental Respiratory Disease, Vancouver Coastal Health Research Institute, University of British Columbia , Vancouver , BC , Canada
[6]Department of Chemical Engineering & Applied Chemistry, University of Toronto, Canada.

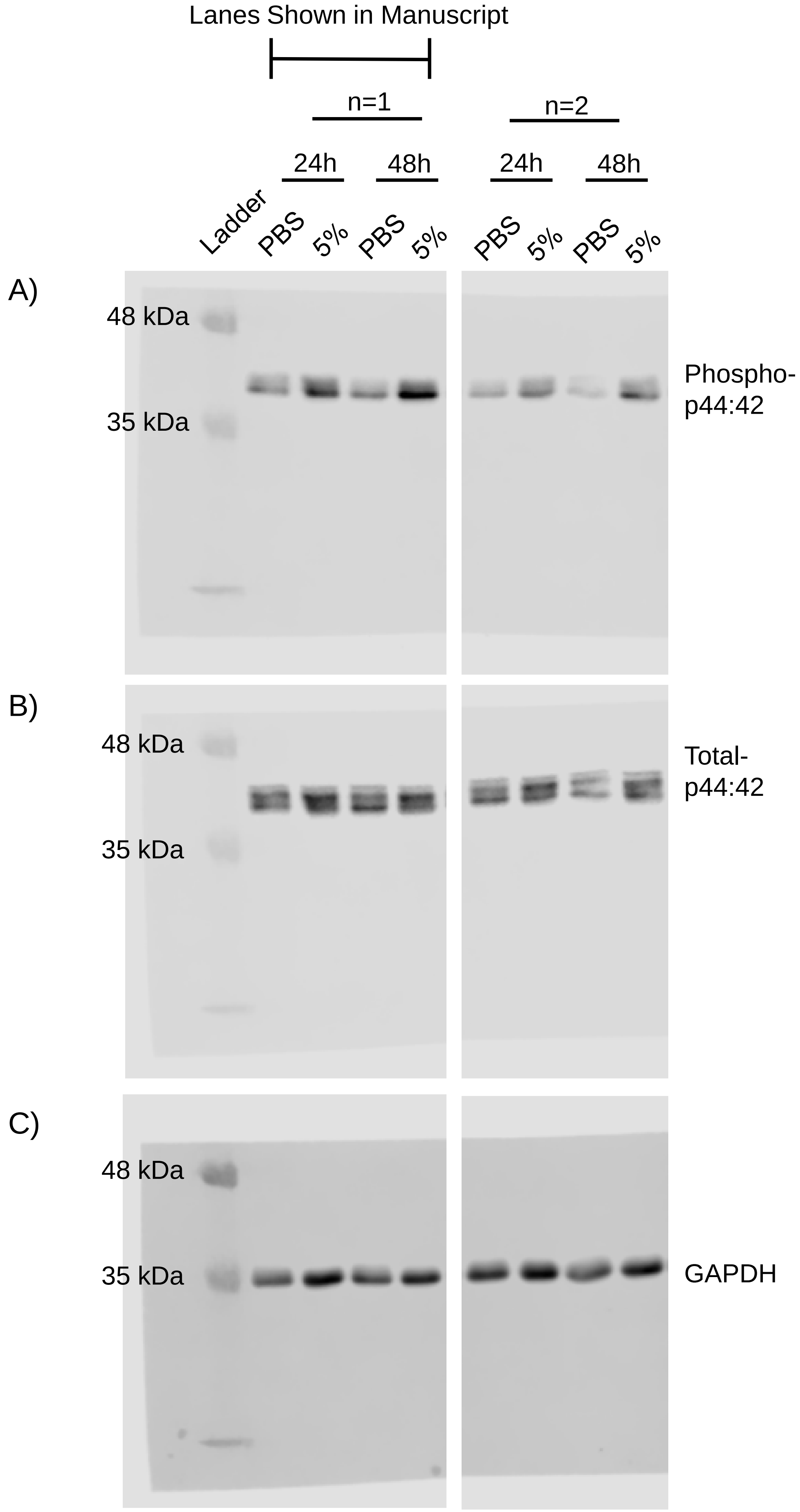

Supplementary Figure S1.

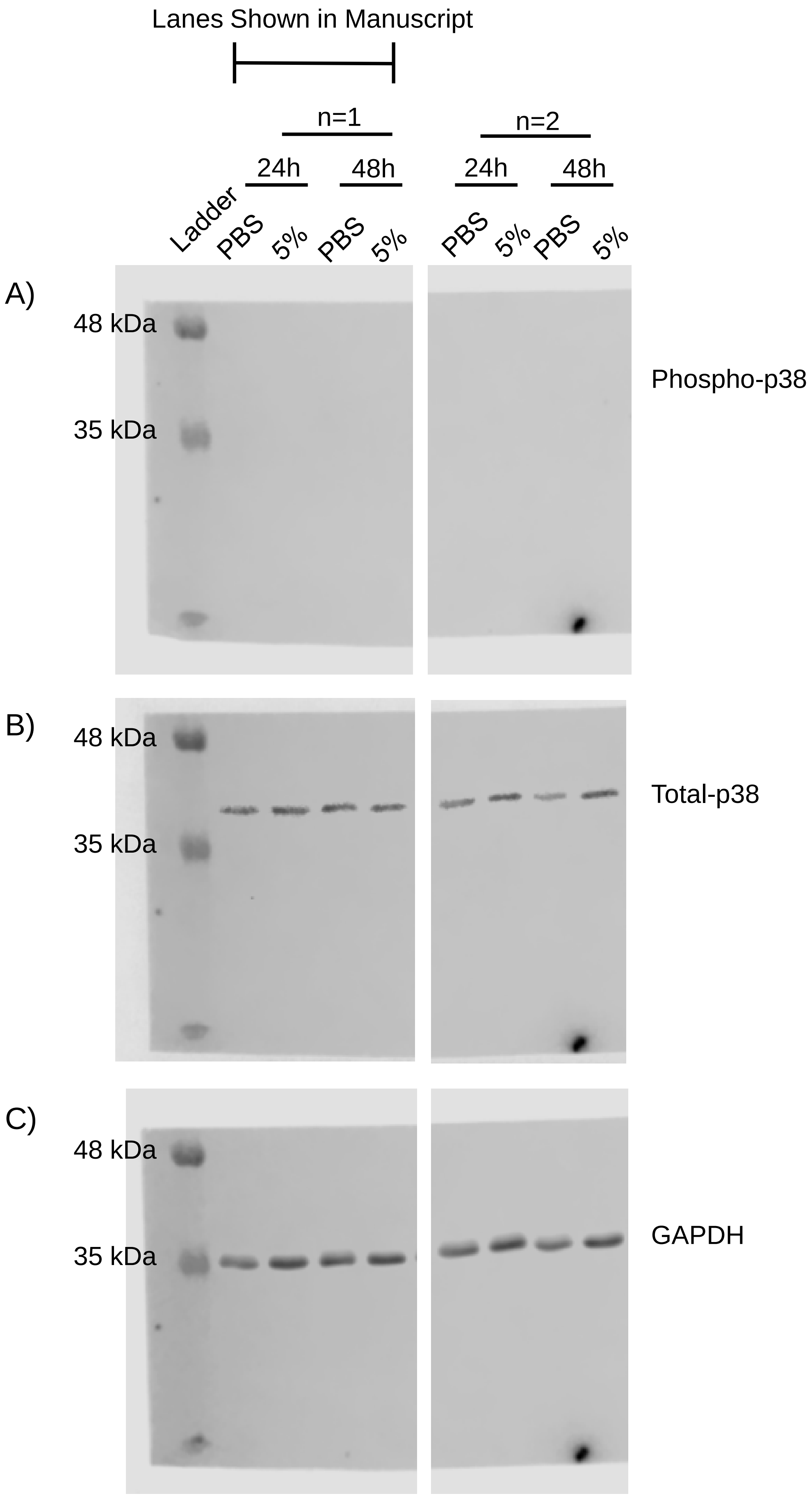

Supplementary Figure S2.

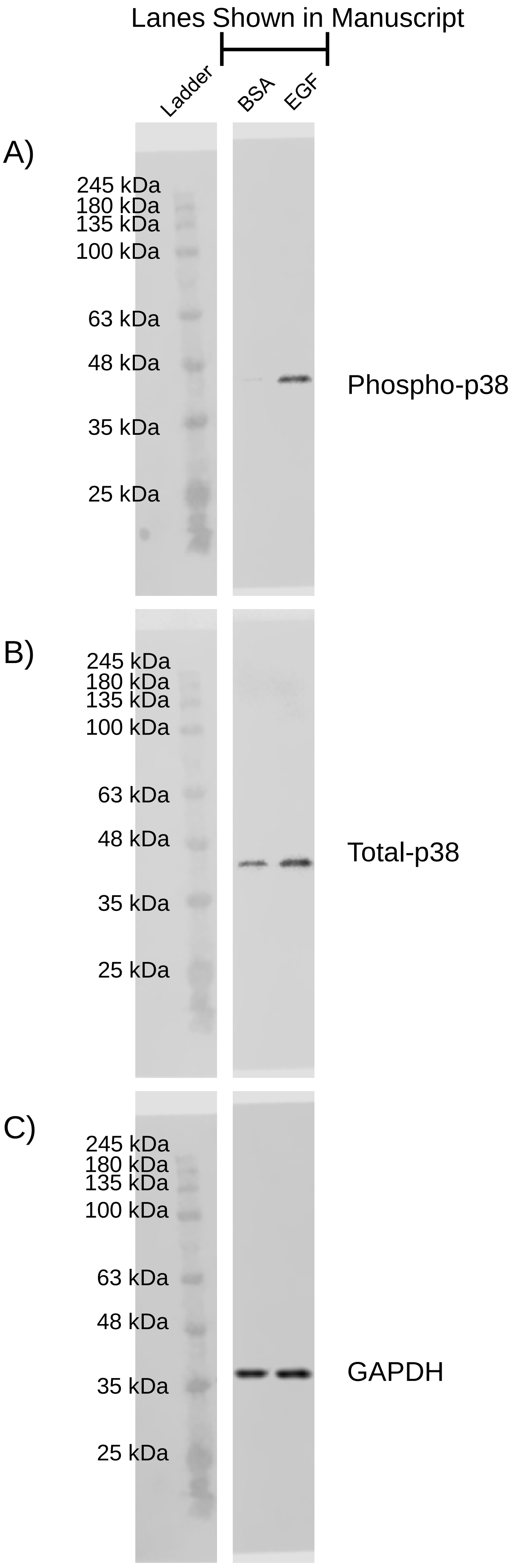

Supplementary Figure S3.

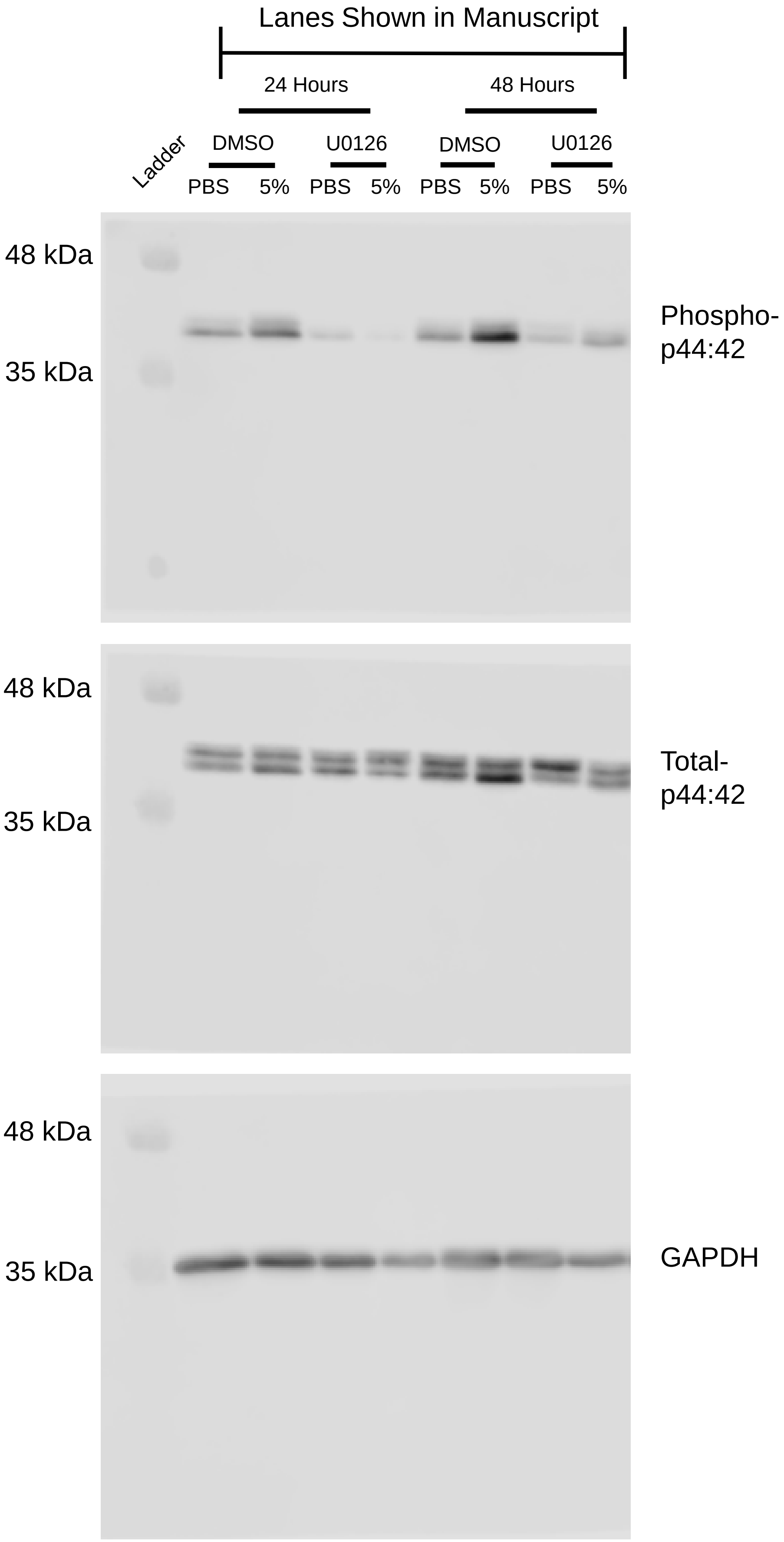

Supplementary Figure S4.
